# Ecological processes underpinning microbial community structure during exposure to subinhibitory level of triclosan

**DOI:** 10.1038/s41598-019-40936-5

**Published:** 2019-03-14

**Authors:** Seungdae Oh, Donggeon Choi, Chang-Jun Cha

**Affiliations:** 1 Department of Civil Engineering, Kyung Hee University, Yongin-si, Gyeonggi-do Republic of Korea; 2 Department of Systems Biotechnology and Center for Antibiotic Resistome, Chung-Ang University, Anseong, Gyeonggi-do Republic of Korea

## Abstract

Ecological processes shaping the structure and diversity of microbial communities are of practical importance for managing the function and resilience of engineered biological ecosystems such as activated sludge processes. This study systematically evaluated the ecological processes acting during continuous exposure to a subinhibitory level of antimicrobial triclosan (TCS) as an environmental stressor. 16S rRNA gene-based community profiling revealed significant perturbations on the community structure and dramatic reduction (by 20–30%) in species diversity/richness compared to those under the control conditions. In addition, community profiling determined the prevalence of the deterministic processes overwhelming the ecological stochasticity. Analysis of both community composition and phenotypes in the TCS-exposed communities suggested the detailed deterministic mechanism: selection of TCS degrading (*Sphingopyxis*) and resistant (*Pseudoxanthomonas*) bacterial populations. The analysis also revealed a significant reduction of core activated sludge members, *Chitinophagaceae* (e.g., *Ferruginibacter*) and *Comamonadaceae* (e.g., *Acidovorax*), potentially affecting ecosystem functions (e.g., floc formation and nutrient removal) directly associated with system performance (i.e., wastewater treatment efficiency and effluent quality). Overall, our study provides new findings that inform the mechanisms underlying the community structure and diversity of activated sludge, which not only advances the current understanding of microbial ecology in activated sludge, but also has practical implications for the design and operation of environmental bioprocesses for treatment of antimicrobial-bearing waste streams.

## Introduction

Triclosan (TCS) is a popular ingredient commonly added to personal care products (e.g., toothpaste, soap, and detergent) and biocides^1–3^. TCS is readily detectable in a variety of environmental settings due to its extensive use, although it has been recently banned in consumer soaps by the US Food and Drug Administration^4^. TCS is ubiquitous in natural aquatic ecosystems and occurs at substantial levels (about hundreds of micrograms per liter) in wastewater-associated environments where it represents a health hazard with presumable ecological risks^1,5^. TCS can also promote broad resistance phenotypes in microorganisms and thus it is possible that it contributes to the spread of antibiotic resistance in the environment^6^. Because a substantial amount of TCS can be carried in urban waste streams (e.g., domestic and hospital/pharmaceutical wastewater), wastewater treatment plants (WWTPs) may represent one of the major sources where TCS can select for antibiotic-resistant bacteria and genes in the environment. Accordingly, many previous studies have recommended appropriate management and monitoring campaigns for TCS in the urban water cycle.

Over the past 100 years, activated sludge processes have been one of the most successful biotechnologies to be widely applied in municipal and industrial WWTPs across the world. Many engineering studies have improved wastewater treatment efficiency and waste management; however, these works primarily focused on manipulating operational parameters of the process, with less attention to the ecology of microbial communities involved. Understanding the assembly patterns of activated sludge microbial communities is essential for predicting the function and resilience of activated sludge ecosystems^7,8^. Recent studies have identified core (highly frequently occurring) bacterial populations across many WWTPs that are thought to carry out key ecosystem functions^9,10^. Microbial interactions can drive deterministic processes governing the dynamics of microbial communities, helping infer the biological performance and functional stability of the ecosystem^9^. Accordingly, advancing our understanding of ecological processes shaping such engineered microbial communities and developing mathematical models that describe these communities are highly desired to better manage system resilience and effluent quality of WWTPs.

In the present study, we addressed the ecological mechanisms involved in controlling community diversity, succession, and function of microbial communities under the presence of TCS as the environmental stressor. Previous studies documented the effects of TCS on microbial communities associated with sink drain biofilms, anaerobic digestion, wetlands, seawater, soil, and gut microbiome (human and fish)^11–18^. Exposure to TCS is known to enrich antibiotic resistance genes (e.g., *tetQ*) in activated sludge^19^; however, the impacts of TCS on the phenotypes and community structure/diversity of activated sludge have not been systematically undertaken. Since the majority of TCS is likely collected into urban WWTPS where high levels of TCS potentially cause ecological disturbances on activated sludge, this study examined activated sludge as a model community in laboratory microcosm studies. The TCS levels tested in this study were related to those from environmentally relevant (hundreds of micrograms per liter) to subinbitory (a few miligrams per liter). A total of 12 16S rRNA gene sequence datasets were analyzed to document time-course changes in phylogenetic diversity and composition in the TCS-exposed communities. In addition to describing the TCS-mediated shifts in community structure, this work conducted time-course physiological characterization of triplicate reactors exposed to TCS, validating the 16S rRNA gene-based findings. Overall, our results provide quantitative assessments on the type and relative importance of ecological processes shaping community assembly largely acting under an environmental stressor exerted by a commonly used antimicrobial. The results of this work will help answer a fundamental question in microbial ecology of activated sludge, which has important implications in the treatment of diverse micropollutants in urban WWTPs.

## Materials and Methods

### Development of activated sludge communities

Activated sludge was sampled from an aeration tank of a local municipal wastewater treatment plant where biological nitrogen removals (nitrification followed by denitrification) occur at aerobic and anoxic tanks. The sludge was inoculated into 1-liter semi-continuous laboratory reactors (600 mL working volume) in triplicate. The substrate (2 g/L of chemical oxygen demand [COD] and 0.6 g/L of salinity) was composed of 1.8 g/L of glucose and the mineral medium solution containing KH_2_PO_4_ (0.34 g), K_2_HPO_4_ (0.6 g), CaCl_2_ (0.05 g), MgSO_4_ 7H_2_O (0.27 g), NH_4_Cl (0.03 g), and FeSO_4_ 7H_2_O (0.09 g) and 10 mL of 100 × trace mineral stock solution (0.35 mg ZnSO_4_ 7H_2_O, 0.21 mg MnSO_4_ H_2_O, 2.1 mg H_3_BO_3_, 1.4 mg CoCl_2_ 2H_2_O, 0.07 mg CuCl_2_ 2H_2_O, 0.14 mg NiSO_4_ 6H_2_O, and 0.21 mg Na_2_MoO_4_ 2H_2_O per liter). One-third of the mixed culture suspension was discarded from the reactor twice a week (i.e., 3.5 days per feeding cycle) and was replaced with the same volume of 2 g COD/L of fresh substrate. The semi-continuous reactors operated in fed-batch mode were aerated (3–4 mg/L dissolved oxygen) and maintained at room temperature (25 °C) and pH 6.9 ± 0.1. After one month of reactor operation, the reactors displayed stable biomass (about 0.4 g/L mixed liquor volatile suspended solids [VSS]) and organic matter removal (>90% COD removal efficiency) every feeding cycle. The operational parameters at steady state were 10.5 days of solid retention time, 0.4 kg COD/kg VSS·d of organic loading rate, and 0.2 kg COD/m·d of food-to-microorganism ratio. After observing the steady state, additional two sets of triplicate batch reactors were further established as subcultures from the control (glucose-fed) cultures by feeding 0.5 mg/L and 5 mg/L of TCS, respectively, in addition to the substrate that was maintained for more than two months. Since TCS occurs at up to 0.08–0.33 mg/L in water environments (e.g., wastewaters and sediment pore waters^5^), 0.5–5 mg/L of TCS tested in this study represented environmental maxima (e.g., highest peaks and/or accidental discharges). Fig. S1 shows the development of activated sludge communities used in this study. Concentrations of VSS, COD, and TCS in the reactor effluents were followed during the entire reactor operation.

### Analytical methods

Triclosan (99% purity) was purchased from Alfa Aesar Inc. (Ward Hill, MA, USA). A 50 g/L TCS stock solution was prepared in acetone and stored at 4°C until use. TCS concentration was measured using a high-performance liquid chromatography (HPLC) system equipped with a Shim-Pack GIST Phenyl, 5 μm, 4.6 × 250 mm column (Shimadzu Asia Pacific Pte Ltd) and a UV-Vis detector. The mobile phase was prepared as follows: 60% acetonitrile and 40% 25 mM NaH_2_PO_4_ buffer adjusted to pH 2.5 with phosphoric acid. The mobile phase was delivered at 1.8 mL/min through the column. The sample of 50 µL volume was injected and detected at the wavelength of 281 nm. The detection limit of TCS using the HPLC system was at 50 µg/L. VSS and COD were measured following standard methods^20^.

### Determining removal routes and kinetics of TCS in activated sludge

To determine presumable routes (e.g., biological vs. abiotic) of TCS removal in the semi-continuous reactors, four sets of triplicate batch experiments were established, separately from the semi-continuous reactors. The four sets were as follows: Abiotic (supplemented with no inoculum + TCS + glucose), Inactive (inactivated inoculum + TCS + glucose), T (inoculum + TCS), and TG (inoculum + TCS + glucose). The inoculum was taken from the mixed culture suspension of the TCS-fed reactors at day 70 and washed twice with 1 × phosphate saline buffer prior to inoculation. The Abiotic condition lacked an inoculum, which was intended to assess the physicochemical losses of TCS such as those via photolysis, hydrolysis, and evaporation. The inactivated inoculum was prepared by autoclaving at 121 °C and 15 psi for 15 min. The Inactive setting included an inoculum that was pre-autoclaved (i.e., no microbial activity intended). The losses of TCS in the Inactive condition could be attributable to sorption to biomass (i.e., biosorption) in addition to those (e.g., photolysis, hydrolysis, and evaporation) of the Abiotic setting. The other experimental conditions (e.g., aeration, temperature, mineral medium, and trace mineral) established in the batch experiments were identical to those of the semi-continuous reactors. The time-course removal from each experimental condition was monitored at 0, 0.5, 1, 2, 4, 8, 12, 16, 24, and 48 h. The removal rate of TCS was calculated by the first-order kinetic equation as follows:

1 $$\mathrm{first}-\mathrm{order}\,{\mathrm{rate}:k}_{{\rm{1}}}=-\frac{\mathrm{ln}({\rm{A}}/{{\rm{A}}}_{0})}{\bigtriangleup t},$$

where A_0_ and A correspond to the initial and time-dependent TCS concentrations, respectively, and ▵*t* is the given time (h).

### Preparing 16S rRNA gene amplicon

The mixed culture suspensions were sampled in triplicate from the control (no TCS added) reactors at days 0 and 42 and from the TCS-exposed communities (fed by glucose plus 5 mg/L of TCS) at day 56 when steady-state performance regarding removal of TCS was demonstrated. Total genomic DNA was extracted from the mixed culture suspension sampled using the MO BIO PowerSoil DNA isolation kit (Carlsbad, CA, USA) according to the manufacturer’s protocol. The concentration and purity of DNA were measured using a NanoDrop One Spectrophotometer (NanoDrop Technologies, Inc.), showing >1 μg/μL and >1.8 absorbance ratios (A260/A280). Total genomic DNA was stored at −20°C prior to sequencing. The sequencing libraries was prepared Macrogen (Seoul, Korea), according to the standard Illumina library protocol. In brief, the DNA quantity was measured by PicoGreen and input gDNA (about 10 ng) was amplified by PCR. The primer sequences used for the first PCR amplifications were as follows: V3-F: 5′-TCGTCGGCAGCGTCAGATGTGTATAAGAGACAGCCTACGGGNGGCWGCAG-3′, V4-R: 5′- GTCTCGTGGGCTCGGAGATGTGTATAAGAGACAGGACTACHVGGGTATCTAATCC-3′. The final purified product was then quantified, according to the Illunima qPCR Quantification Protocol Guide (Illumina, San Diego, USA) and qualified using the TapeStation DNA screentape (Agilent Technologies, Waldbronn, Germany). The paired-end (2 × 300 bp) sequencing was performed by Macrogen using the MiSeq™ platform (Illumina, San Diego, USA).

### 16S rRNA gene sequence data analysis

The 16S rRNA gene sequence was analyzed according to the MiSeq SOP pipeline^21^. The commands and parameters used for sequence analysis were described previously^22,23^. In brief, raw sequences were filtered using the following parameters: maxambig = 0, minimum length = 200, maximum length of homopolymer = 8, and all other parameters at their default settings. High quality sequences after preprocessing were chimera-checked using chimera.vsearch with the default settings, after which the remaining sequences were taxonomically classified using classify.seqs with a cut of 80. Sequences taxonomically assigned to chloroplasts, mitochondria, unknown, archaea, and eukaryotes were excluded. The remaining sequences were clustered into representative operational taxonomic units (OTUs) based on a 97% nucleotide identity cutoff, using dist.seqs with a cutoff of 0.03 and cluster with the default settings. The Mann-Whitney U test was employed to assess differential community characteristics (e.g., alpha diversity index, community composition, and phenotype) across communities.

### Nucleotide sequence accession number

The 16S rRNA gene sequence datasets used in this study were deposited in GenBank under the following accession numbers: Control_0_1_ (SRS2340183), Control_0_2_ (SRS2340176), Control_0_3_ (SRS2340220), Control_42_1_ (SRS2340175), Control_42_2_ (SRS2340198), Control_42_3_ (SRS2340197), TCS_3.5_1_ (SRS2340205), TCS_3.5_2_ (SRS2340170), TCS_3.5_3_ (SRS2340172), TCS_56_1_ (SRS2340171), TCS_56_2_ (SRS2340173), and TCS_56_3_ (SRS2340174).

## Results and Discussion

### Shifts in the community structure and diversity of activated sludge under TCS exposure

**Figure 1:**
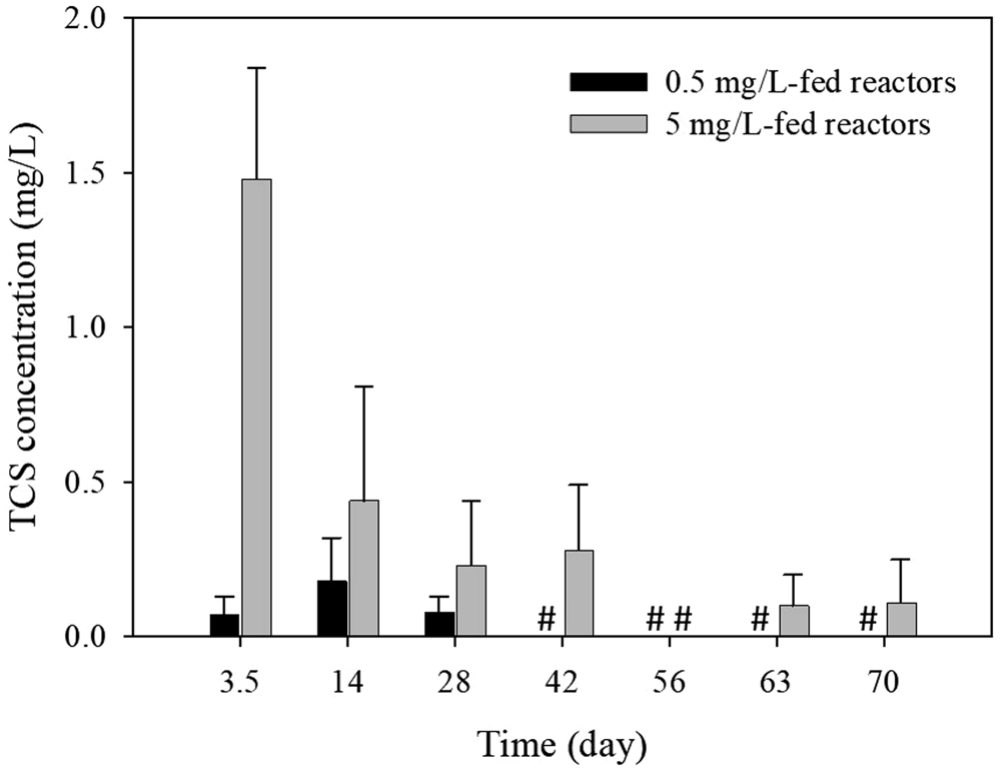
Performance of bioreactors. TCS concentrations in the effluents of semi-continuous bioreactors over time. The error bars indicate the standard deviation from the mean. COD removal rates (85–90%) and mixed liquor VSS (0.41–0.45 g/L) of the 5 mg/L-fed reactors at steady state at days 42–70 were similar to those (88–92% and 0.42–0.46 g/L, respectively) of the control reactors. ^#^Below the detection limit (<50 μg/L).

Figure 1 shows the fate of TCS in activated sludge bioreactors fed by 0.5 and 5 mg/L of TCS, respectively, over 70 days. The TCS levels in the effluents of the 0.5 mg/L-fed reactors were less than the detection limit (50 µg/L) at days 42–70. The 5 mg/L-fed reactors showed relatively poor removals (70%) in the first cycle and gradually increased the removals: 91% at day 14, 94% at day 28, and > 95% at days 42–70. Profound change of the TCS removal phenotypes was observed in the 5 mg/L-fed communities, which led us to detail the potential TCS-mediated shifts in the 5 mg/L-fed microbial communities.

**Figure 2:**
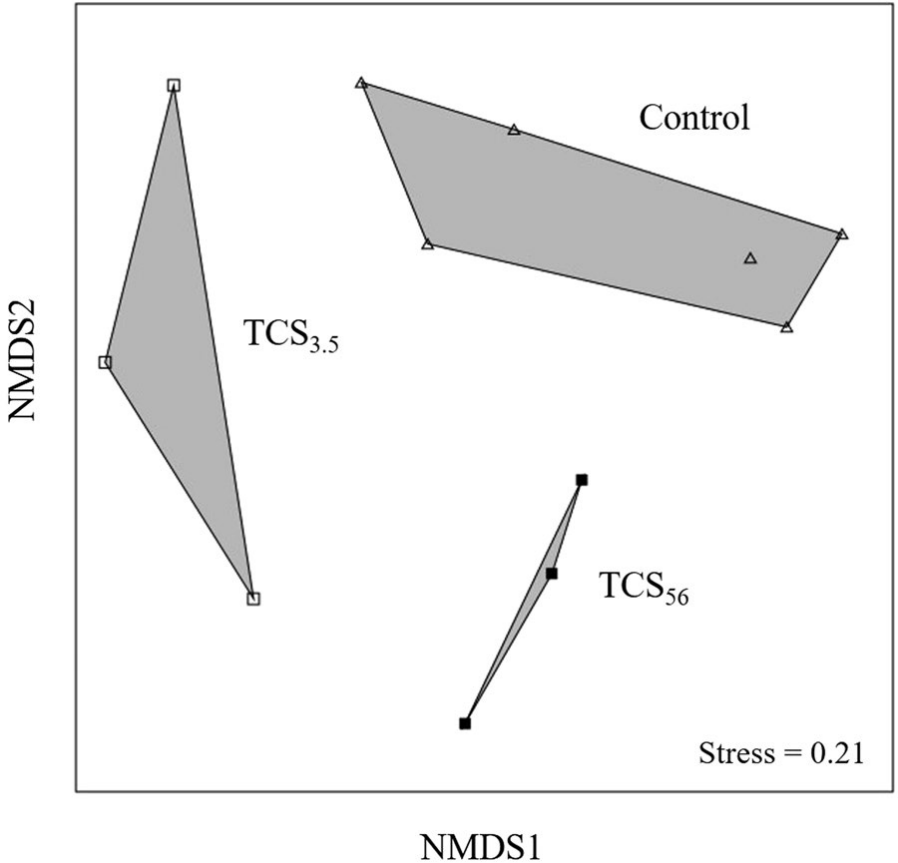
Non-metric multidimensional scaling (NMDS) ordination of activated sludge communities. The NMDS plot was generated based on OTU composition data using the Bray-Curtis distance metric. Triangle marks represent the control communities. Open and closed squares represent TCS-exposed communities obtained at day 3.5 and 56, respectively.

To document the time-course community dynamics, triplicate 16S rRNA gene sequence datasets were obtained from the 5 mg/L TCS-exposed reactors (fed both TCS and glucose) at day 3.5 (TCS_3.5_, sampled at the end of the first feeding cycle) and day 56 (TCS_56_, a typical cycle at steady state). To assess the degree of random variability in the community structure and diversity, triplicate 16S rRNA gene datasets were also obtained from the control reactors (fed by glucose) at days 0 and 42. MiSeq sequencing generated a total of 40,000–64,000 sequences per sample. The total number of OTUs of the 12 16S rRNA gene datasets ranged from 150 to 300 per sample (i.e., estimated by rarefication to 40,000 sequences for estimating the number of OTUs and alpha diversity indices). The OTU composition data across datasets were used to perform non-metric multidimensional scaling (NMDS) analysis (Fig. 2). The NMDS plot shows that communities closely clustered within each group and were distinguishable from each other group in the ordination. The pairwise community similarity using the Bray-Curtis distance metric was measured at 0.15 ± 0.09 (control vs. TCS_3.5_) and 0.17 ± 0.03 (control vs. TCS_56_). A permutational multivariate analysis of variance (PERMANOVA) test^24^ indicated significant difference in phylogenetic structure among the three communities (Bonferroni-corrected *p* < 0.05). These results suggest that exposure to TCS could result in significant alteration of the community structure under TCS exposure even in a short period (3.5 days).

Ecological processes that can affect the structure and diversity of microbial communities include, but not limited to, diversification, selection, dispersal, and ecological drift. Diversification, dispersal, and ecological drift are considered stochastic processes (central to neutral theory), while selection is a deterministic process^25^. Environmental stressors such as pH, salinity, and temperature can be deterministic factors driving the community assembly. Many studies have shown changes in microbial community assembly that are not necessarily deterministic but instead stochastic^25,26^. Ecological stochasticity (e.g., random changes in community assembly and function due to the stochasticity of taxa in birth, death, and competition) can create significant variability in phylogenetic structure (i.e., beta diversity) across communities even under homogeneous environmental conditions. Our quantitative assessments on the strength of ecological processes showed that the deterministic factor (TCS-mediated alteration in community structure) overwhelmed (Bonferroni-corrected *p* < 0.05) the stochastic (reflected from random variability within six control reactors). Further, the intra-group community structure under TCS exposure was similar in TCS_3.5_ (0.65 ± 0.41 within group) and TCS_56_ (0.79 ± 0.25 within group). In contrast, there was substantial temporal variation (0.57 ± 0.36 within group) generated within triplicate control reactors, indicating considerable stochastic variation, although they were strictly controlled under identical laboratory settings. Accordingly, for future studies investigating the degree of consequences of a deterministic factor on community structure, we strongly recommend considering the substantial stochastic variability in the structure of replicate communities generated even under a homogeneous laboratory condition, the relative importance of which many previous studies have ignored. While many studies have investigated the deterministic consequences in microbial community structure by varying operational conditions and environmental factors, considering the random temporal changes created in the control condition for the same period of operation with treatment (i.e., measured for 42 days as in this study) will help more accurately estimate (and not overestimate) the significance of a deterministic factor relative to ecological stochasticity.

**Figure 3:**
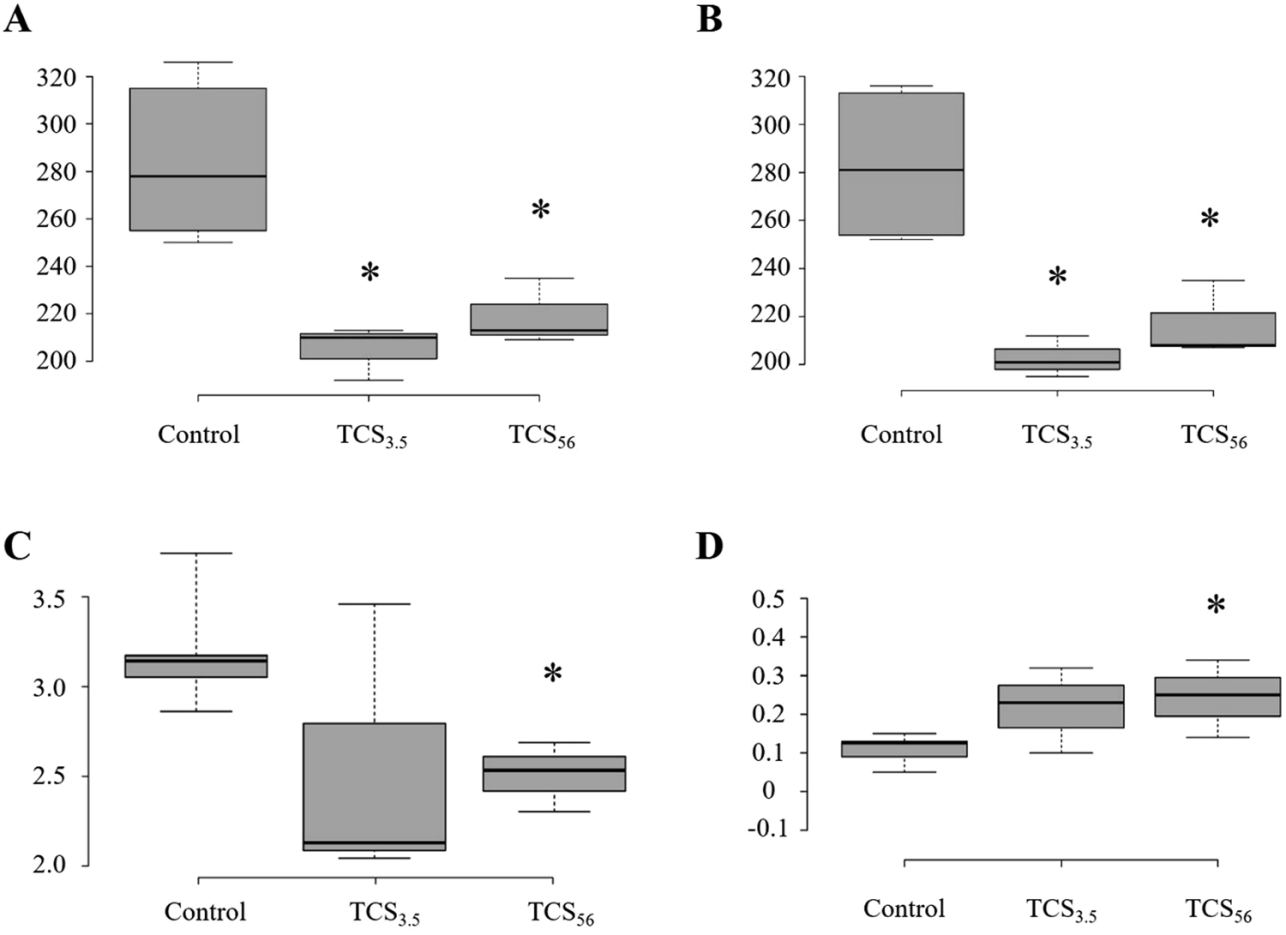
Alpha-diversity indices of both control and TCS-exposed communities. Box plots depict the medians (the central horizontal line), inter-quartile ranges (boxes), and 95% confidence intervals (whiskers). Species richness is shown with the Chao1 (**A**) and Ace (**B**) indices. Species diversity is represented by the Shannon (**C**) and Simpson (**D**) indices. Asterisks indicate statistically significant differences (*p* < 0.05) between the control and TCS-exposed communities.

The TCS-mediated shifts in the community phylogenetic structure (beta diversity) motivated us to examine the effects of TCS on alpha diversity indices among the communities. The number of OTUs found in the control communities was 247 ± 31. The number decreased to 177 ± 17 (TCS_3.5_) and 188 ± 3 (TCS_56_), suggesting dramatic reduction by more than 30%. The alpha diversity indices^21^, representing species diversity (Shannon and Simpson) and richness (Chao1 and Ace), are shown in Fig. 3. The average Chao1 and Ace indices of the control communities were 283 ± 32 and 283 ± 29, respectively, and were dramatically reduced (*p* < 0.05) in both TCS-exposed communities by more than 20%. The Shannon index was 3.20 ± 0.29 in the control communities and decreased to 2.55 ± 0.83 (*p* = 0.11) in the TCS_3.5_ and 2.51 ± 0.20 (*p* < 0.05) in the TCS_56_, although the *p*-value between the control and TCS_3.5_ did not reach significance. Similar results (significant reduction in species diversity) were obtained based on the Simpson indices. Overall, both short-term (3.5 days) and long-term (56 days) exposure to TCS significantly decreased species richness, while long-term exposure could pose more pronounced perturbations, reflected in the species diversity indices.

Microbial community diversity can be represented by the number (richness), diversity, and equitability (evenness) of taxa. Our results clearly show that both species richness and diversity were dramatically reduced (i.e., species richness by 28% and diversity by 22%) in response to TCS. While there are many alpha diversity metrics, no index is without bias, and each metric has its own strengths and weaknesses^27^. Since the results of at least two frequently used methods for each index were highly consistent, our conclusions of significant reduction in species richness and diversity caused by TCS are highly robust. A large body of literature reports that high diversity is associated with functional diversity and ecosystem stability^27–30^. Although high diversity might be considered a desirable outcome for microbial communities, other studies have shown examples where high diversity is not necessarily beneficial. A recent work suggested^27^ that the results of alpha diversity together with beta diversity are not the ecological processes themselves but the outcomes. In this light, we used the results (Figs 2 and 3) to establish a basis for exploring the underlying mechanism resulting in the outcome of an ecological process, which is to be addressed in the following section.

### Ecological process controlling structure and diversity: Selection of TCS degraders

**Figure 4:**
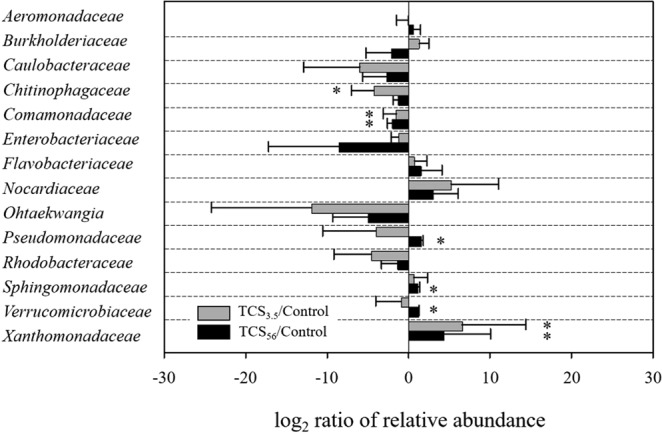
Log_2_-transformed relative family abundance. The major families were selected considering the average abundance (>1%). The logarithm of the relative abundance ratio (i.e., TCS-exposed divided by the control) in base 2. The error bars indicate the standard deviation of the mean. Asterisks indicate differences (*p* < 0.05) in relative abundance of a family between the control and TCS-exposed communities.

**Figure 5:**
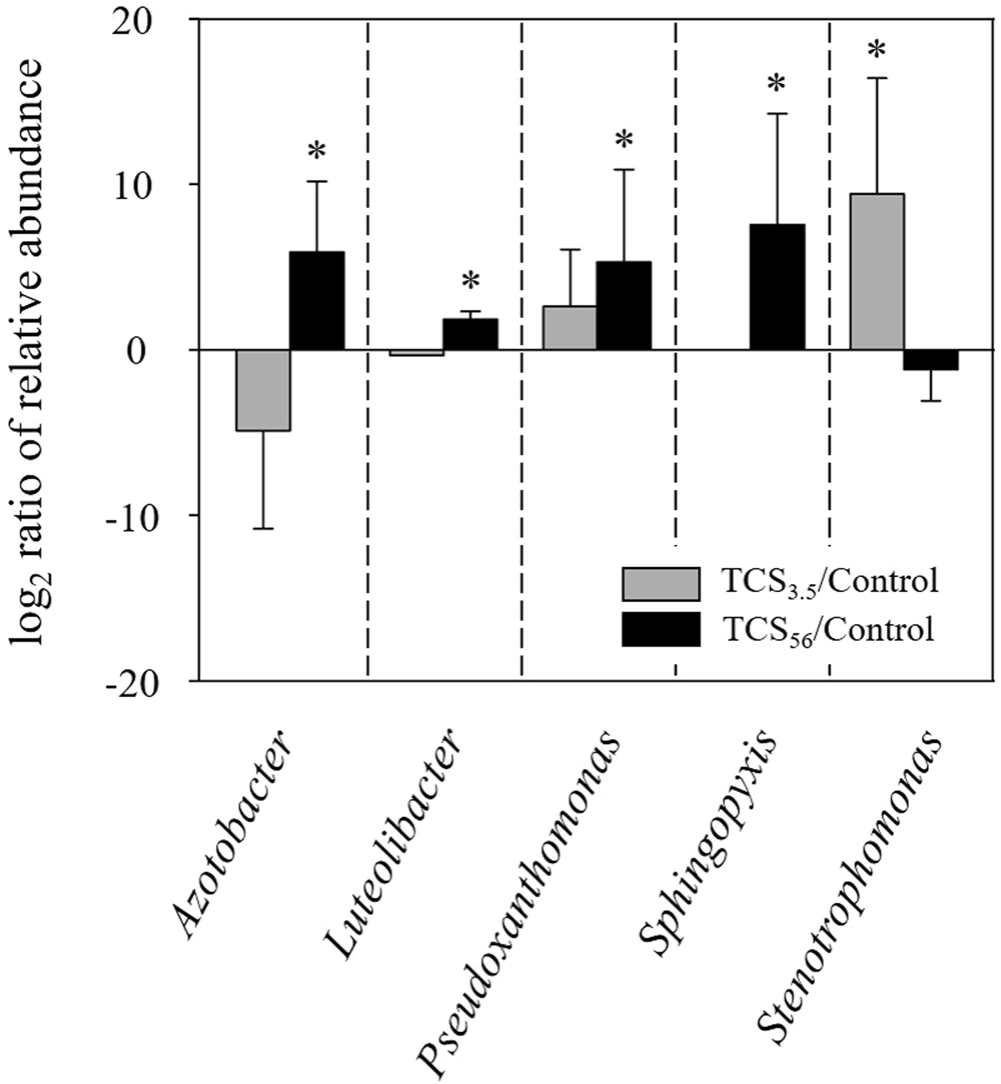
Log_2_-transformed relative abundance of select genera. Genera with differential abundance between TCS-exposed and control communities are shown. Asterisks indicate significantly different abundance (*p* < 0.05) between the control and TCS-exposed communities (TCS_3.5_ vs. Control or TCS_5_ vs. Control).

Taxonomic analysis using the MOTUHR pipeline identified 14 major families among the three communities. Figure 4 shows the differential relative abundance of the major families in the TCS-exposed communities compared to the control. Among others, *Xanthomonadaceae, Pseudomonadaceae, Sphingomonadaceae*, and *Verrucomicrobiaceae* were significantly enriched through exposure to TCS, among which *Xanthomonadaceae* was selected even within the first feeding cycle. The community composition at the genus level showed significant enrichment of five genera in the TCS-exposed communities (Fig. 5). While *Stenotrophomonas* was overrepresented in TCS_3.5_ compared to the control communities, 56 days of TCS exposure selected for *Sphingopyxis* (8-fold change, i.e., a change to 8 times), *Pseudoxanthomonas* (5-fold), *Luteolibacter* (2-fold), and *Azotobacter* (6-fold).

**Figure 6:**
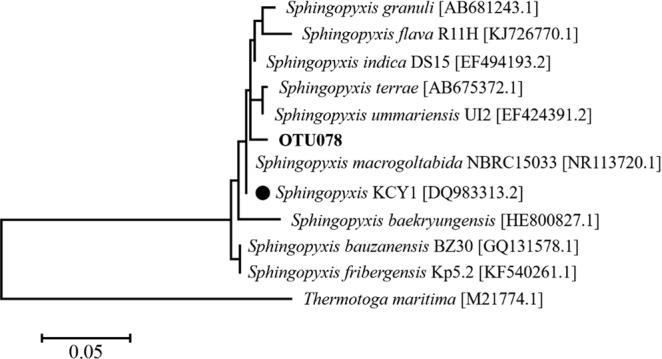
Phylogenetic tree of *Sphingopyxis* populations. The phylogenetic tree was constructed using the Maximum Likelihood method based on the Tamura-Nei model. The reference *Sphingopyxis* sequences were from the NCBI 16S ribosomal RNA sequences. A black circle indicates the previously described strain with demonstrated TCS catabolic capacity, originating from wastewater (Lee *et al*. 2012a). Accession numbers are provided in brackets.

Since our experiments were conducted under identical conditions with no invasion/movement of new organisms on a relatively short time scale, we speculated that selection of organisms due to fitness differences, rather than other mechanisms such as dispersal and diversification, might prevail under the selective pressure (TCS exposure). TCS selected for *Sphingopyxis*, which are aerobic alphaproteobacteria capable of accumulating poly-β-hydroxyalkanoates. Although the exact role in activated sludge remains unclear, *Sphingopyxis* are known to detoxify many xenobiotics such as TCS and chlorophenol. In particular, a *Sphingopyxis* population (strain KCY1) originating from wastewater could degrade TCS at a wide range of concentrations (0.005–5 mg TCS/L)^31^. The 16S rRNA gene data analysis identified one OTU sequence (OTU078) phylogenetically affiliated with *Sphingopyxis* (Fig. 6). OTU078 showed more than 97% sequence similarity to 10 representative *Sphingopyxis* sequences deposited in the NCBI 16S ribosomal RNA sequence database. Of note was the close phylogenetic relationship (98.9% similarity) between OTU078 and KCY1 with the demonstrated TCS metabolic capacity.

**Figure 7:**
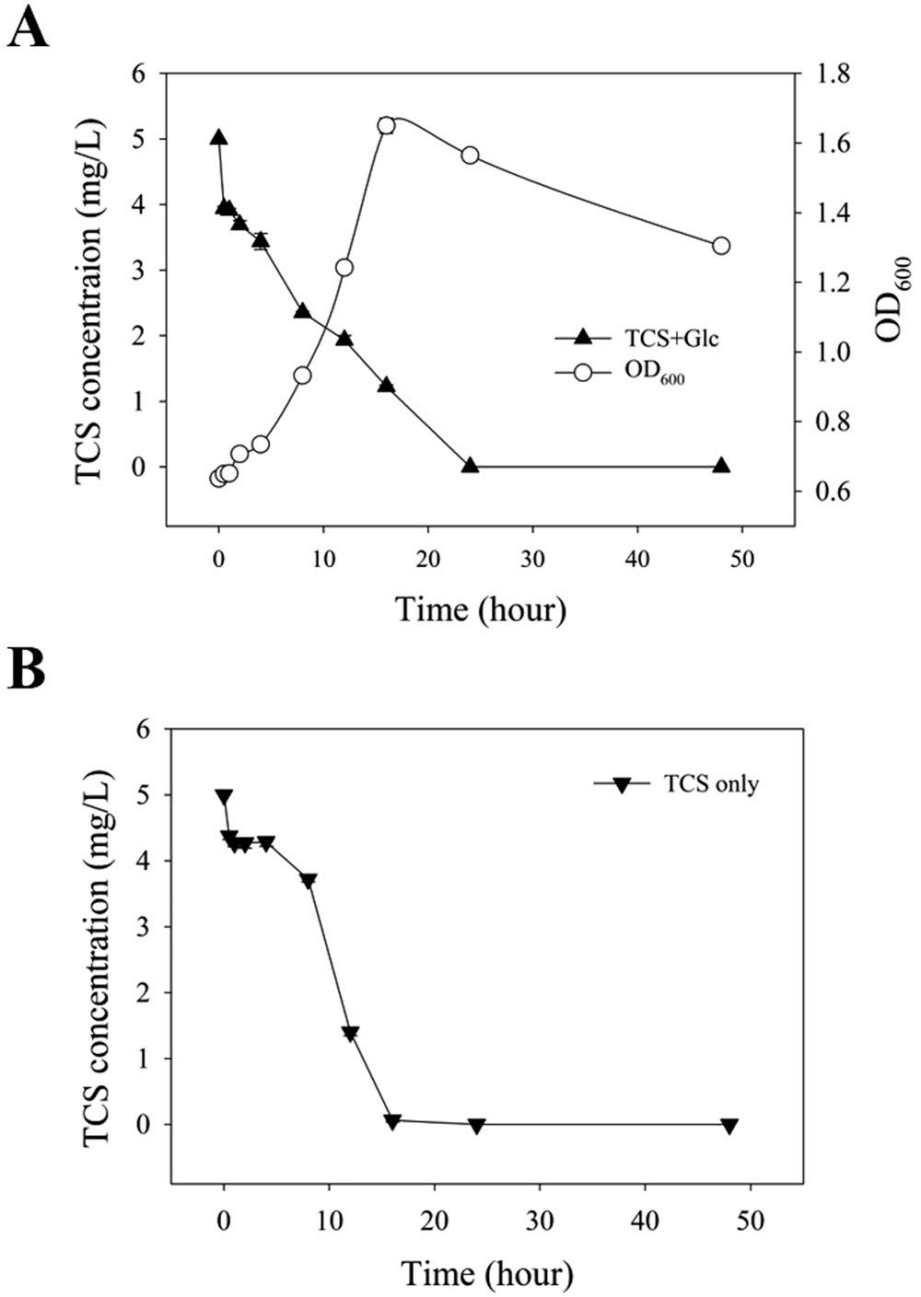
Time-course of TCS removal observed in batch experiments using inocula taken from semi-continuous bioreactors at steady state. (**A**) Cell growth and TCS removal profile in the TG condition (See Materials and Methods for more details in the experimental settings) where dual carbon sources were supplemented. (**B**) Time-course of TCS removal in the T condition where TCS was used as a sole carbon source.

The 16S rRNA gene-based community profiling results (i.e., selective enrichment of *Sphingopyxis* under exposure to TCS) were highly consistent with the physiological characterization results (i.e., gradual increase of the time course TCS removal rates observed in Fig. 1). The almost complete removal of TCS observed at steady state (Fig. 1) further called for an analysis of TCS removal routes occurring in the reactors. The mixed culture suspension was sampled from the semi-continuous reactors (TCS-fed) at day 56 and subjected to separate batch tests with several experimental settings. The TG batch test (with active inoculum) fed by dual carbon sources (glucose plus TCS) showed complete transformation of TCS within 24 hours, while the majority of the breakdown occurred during exponential growth (represented by OD_600_) (Fig. 7A). The level of TCS did not significantly change in the Abiotic setting (i.e., without inoculum) (Fig. S2). In the Inactive setting (with inactivated inoculum [heat-killed] using autoclave), 58% of the TCS quickly disappeared (within 30 minutes), and the remaining fraction did not change significantly for the remaining 48 hours (Fig. S2). These results suggest that the TCS removal observed in the Inactive setting could be attributable to abiotic pathways, in particular, via adsorption rather than other routes (e.g., volatilization, photolysis, and hydrolysis). Further, the fact that adsorption-mediated removal could not account for the complete removal of TCS observed in Fig. 7A suggests that biodegradation could be another primary route of TCS removal, as reported previously^5,31,32^. Further, Fig. 7B shows complete removal of TCS within 24 hours by the communities in the T setting fed by TCS as a sole carbon source (no glucose added), demonstrating the metabolic capacity for TCS by the *Sphingopyxis*-enriched community via direct metabolism, not necessarily co-metabolic.

Previous studies have shown that several bacteria originating from soil and wastewater sources have TCS metabolic capacity at 0.2–5 mg/L, including *Pseudomonas, Alcaligenes, Nitrosomonas*, and *Sphingomonas* as well as *Sphingopyxis*^31–34^*. Pseudomonas* and *Alcaligenes* carry out dioxygenase-mediated catabolic pathways on halogenated phenolic compounds and are able to grow by utilizing TCS as a sole carbon source^33^. *Sphingopyxis* and *Sphingomonas* break down TCS via a meta-cleavage pathway using 2,3-dioxygenase^31,34^. In addition to *Sphingopyxis*, 16S rRNA gene data analysis revealed the abundance of two taxa, *Pseudomonas* (0.01 ± 0.02%) and *Sphingomonas* (0.07 ± 0.07%), in the control communities, whose TCS degradation activities were previously reported. However, why *Sphingopyxis* could be selectively enriched under the TCS exposure and the others were not enriched remains a question. It should be noted that the TCS utilization rates of bacterial populations reported in the literature are very different^31–34^, indicating that the differential TCS-utilizing activity might confer a different degree of competitive fitness for succession under TCS exposure. Of note was higher first order degradation rate constants (>8 × 10^−2^/h) of two *Sphingomonadaceae* populations (*Sphingopyxis* and *Sphingomonas*) on TCS utilization^31,34^ compared to those (<3 × 10^−2^/h) of *Pseudomonas*^33^. These previous estimates support more successful succession (competition) of the *Sphingopyxis* population than *Pseudomonas* that was observed during our 16S rRNA gene sequencing and analysis (Figs 4 and 5).

Next, we calculated the TCS degradation kinetics of the *Sphingopyxis*-enriched communities using the data shown in Fig. 7B (i.e., at the condition metabolizing TCS as the sole carbon source). The TCS utilization rate (33.9 × 10^−2^/h) observed in our study was higher than other previous estimates ( < 10 × 10^−2^/h) of TCS degradation by activated sludge at similar levels (1–5 mg/L of TCS)^32,35,36^. Accordingly, the higher TCS utilization rate observed in our study was likely due to the microbial community adaptation (associated with the increase of TCS removal) that occurred within the first month of reactor operation (Fig. 1). However, since the community-level TCS utilization rate was higher than the previously characterized estimate of *Sphingopyxis*, it could not rule out the potential involvement of other bacteria in the degradation of TCS observed in Fig. 7. Nevertheless, the enhancement of TCS removal rate observed in Fig. 1 was, at least in part, attributable to selection of *Sphingopyxis* with a high TCS utilizing capacity. Since little information is available on *Sphingopyxis* related to TCS degradation, further investigation on the isolation and physiological/genomic characterization of *Sphingopyxis* isolates will be necessary to help better predict the fate of TCS in engineered biological systems such as WWTPs.

*Azotobacter* is a genus of *Pseudomonadaceae* that is often found in industrial wastewaters and soils contaminated with antibiotics, pesticides, and heavy metals^37,38^. *Azotobacter* are closely related (phylogenetically and phenotypically) to genus *Pseudomonas* that is capable of degrading TCS^33,39,40^. Since *Azotobacter* carries an array of diverse oxygenase genes (e.g., catechol 2,3-dioxygenase and ammonia monooxygenase) potentially associated with enzymatic degradation of TCS^31^, these organisms might be involved in metabolism of TCS and thus were potentially selected upon TCS exposure, although this argument awaits further experimental examination.

### Ecological process controlling structure and diversity: Selection of potential antimicrobial resistant bacteria

Exposure to TCS could select for *Pseudomoxanthomonas* and *Luteolibacter*, in addition to the demonstrated (*Sphingopyxis*) and potential (*Azotobacter*) TCS degraders (Fig. 5). The environmental stressor (TCS exposure) is thought to exert varying levels of environmental filtering to organisms, conferring fitness differences for survival, growth, and reproduction of the organisms. While bacteria can resist a variety of antimicrobials to a certain extent, the inherent functional traits resisting TCS could be another driver facilitating the shift in activated sludge structure in response to TCS. *Pseudoxanthomonas* are gram negative, non-spore forming bacteria, phylogenetically closely related to *Stenotrophomonas* and *Xanthomonas*. These organisms are often isolated from environments contaminated with oil, where they are supposed to play a key role in degradation of a variety of hydrocarbons including benzene, toluene, and ethyl-benzene^41,42^. A denaturing gradient gel electrophoresis analysis reported that sink drain biofilm communities enrich *Pseudoxanthomonas* following long-term exposure to TCS-containing detergent^11^. *Luteolibacter* were particularly selected in gut microbiota of infants whose families use TCS-containing personal care products^16^, suggesting the need for further testing of personal care products on human microbiome and health. These previous studies support the potential inherent/adaptive resistance phenotypes of the two taxa*, Pseudomoxanthomonas* and *Luteolibacter*, against TCS.

**Figure 8:**
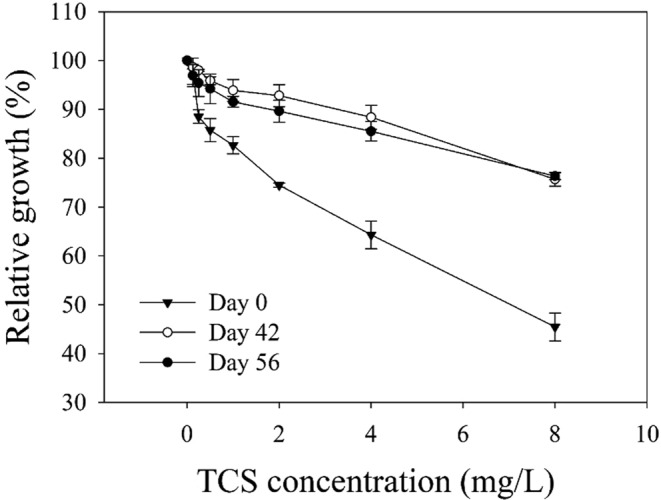
Relative growth of communities at various TCS levels. TCS susceptibility was determined as described previously (Oh *et al*. 2013) by measuring the relative growth at a range of TCS concentrations (0.125–8 mg/L) compared to those without TCS. The OD_600_ of each sample after 24 hours of incubation was normalized to that of control samples (no TCS added). Testing was performed at day 0 (before TCS exposure) and during exposure to TCS (at day 42 and 56, respectively).

The extent of antimicrobial resistance susceptibility of the activated sludge communities in response to TCS exposure was examined over time at days 0, 42, and 56 (Fig. 8). TCS susceptibility was determined by comparing the cell growth with TCS to those without TCS (i.e., control)^29^. After 24 hours of incubation, the cell growth (i.e., the OD_600_ data measured using a spectrophotometer) with 0.125–8 mg/L of TCS was normalized to that of the control to provide the relative cell growth, respectively. The relative community growth at day 0 was inhibited by 88–45% at 0.25–8 mg/L TCS. In contrast, the relative growth at the same level of TCS was 95–80% compared to the control at days 42–56, showing significant reduction (*p* < 0.05) in susceptibility to TCS in the tested range (0.25–8 mg/L). These results corroborated the significant increase of antimicrobial resistance phenotypes upon exposure to TCS, which is consistent with the selection of potential TCS-resistant bacterial populations observed in Figs 4 and 5. Although the selection of *Pseudoxanthomonas* and *Luteolibacter* might partly account for the increased resistance against TCS, other bacterial populations might also acquire broad-spectrum resistance phenotypes through adaptive evolution via point mutations and/or horizontal gene transfer of multi-drug resistant genes under TCS exposure. Our recent works showed good examples of how omics methods can be used to identify genetic mechanisms of community members involved in degradation of and resistance to toxic xenobiotics^29,43^. We are currently employing omics technologies (metagenomic and metatranscriptomic analyses) with a particular focus on elucidating the genomic elements responsible for the induction of antimicrobial resistance to TCS, as observed in this study.

### Ecological processes controlling structure and diversity: Reduction of core bacterial members

*Chitinophagaceae* and *Comamonadaceae* were significantly decreased (*p* < 0.05) among the 14 major families (Fig. 4). The average relative abundance of *Chitinophagaceae* was 0.3 ± 0.3% (TCS_3.5_) and 2.0 ± 1.4% (TCS_56_), which was significantly reduced from that of the control (5.0 ± 2.2%). *Comamonadaceae* also showed a significant reduction in response to TCS at days 3.5 (1.0 ± 0.2%) and 56 (0.7 ± 0.5%) compared to the control (2.9 ± 0.7%). *Chitinophagaceae* and *Comamonadaceae* represent core bacterial members of activated sludge that frequently occur across many full-scale, geographically differentially located WWTPs and are found to be relatively sensitive to antimicrobials^10,44^. Members of the *Chitinophagaceae* family dominating activated sludge are aerobic heterotrophs capable of degrading organic matter and are involved in biosynthesizing and exporting extracellular polymeric substances (EPSs)^45^. Bacteria can aggregate to form biofilms, flocs, and granules in activated sludge via physical interactions such as adhesion and cohesion. Bacterial EPSs can initiate the attachment of cells to solid particles and are essential for structural integrity by helping cells establish and colonize biofilms of the particulate supports. EPSs account for 50–90% of the total organic matter in biofilms and are essential for the formation of activated sludge flocs^46^. Floc formation is of great practical importance because it can significantly affect the performance of many unit processes (e.g., flocculation, settling, and dewatering) in WWTPs. In addition, EPSs can act as protective barriers for biofilm cells against harmful environmental perturbations (e.g., toxic compounds and changes in temperature and pH). The surface properties (hydrophilicity/hydrophobicity) of EPSs offer additional benefits for contaminant removal by adsorption of diverse metals and organic/inorganic compounds^46,47^. On the other hand, *Comamonadaceae* are often associated with nitrogen removal in activated sludge. Some members of the *Comamonadaceae* family (e.g., *Acidovorax* and *Comammonas*) are acetate-utilizing denitrifiers and are predominant in activated sludge^48,49^. Fig. S3 shows the relative abundance of genera that were found in the reactors and that were affiliated to *Chitinophagaceae* and *Comamonadaceae*, respectively. In addition to the selective decrease of *Acidovorax* phenotypically related to denitrification aforementioned, *Ferruginibacter*, a genus of *Chitinophagaceae*, were found to be significantly reduced under exposure to TCS. Previous works reported that *Ferruginibacter* are highly enriched in activated sludge and potentially associated with bioflocculation and biofilms^50–52^, consistently supporting the suggested role in floc formation. In sum, the significant reduction in core activated sludge members observed in this study highlighted the potential disruptions on the ecosystem functions of activated sludge directly associated with process performance (e.g., wastewater effluent quality and sludge processing), which therefore suggests future experiments assessing the effects of TCS on biofilm formation and nutrient removal.

This work quantitatively assessed microbial community dynamics during exposure to a high level of TCS (5 mg/L). Although the toxicity threshold of TCS can be variable across organisms, a few milligrams per liter (or higher) of TCS pose perturbations to the functions of activated sludge ecosystems. For example, our data suggested that 50% of cell growth activities of activated sludge communities were reduced between 4 and 8 mg/L of TCS. The results of this study were in a good agreement with a previous study reporting that about 2 mg/L of TCS is 50%-inhibitory to activated sludge (measured with biochemical oxygen demand consumption activities). The previous work highlighted that activated sludge could resist against TCS much more than other aquatic organisms (e.g., algae) by a factor of 5–1800. Accordingly, the TCS-mediated shifts in microbial community diversity and structure systematically described in this study were relevant to those at subinhibitory levels of TCS capable of disrupting important ecosystem functions of activated sludge such as oxygen uptake and biomass growth activities.

## Conclusions

Time course community profiling using 16S rRNA gene sequencing revealed the importance of the deterministic ecological process relative to the stochastic for controlling the structure and diversity of activated sludge communities under exposure to subinhibitory levels of TCS. The results of this work provided not only quantitative estimates on the consequence of TCS exposure to community structure and diversity, but also the mechanism of the ecological process mediating the deterministic shifts in the community assembly (e.g., selection of TCS degrading/resistant bacteria and reduction of core activated sludge members). Physiological characterization (e.g., TCS biodegradation activity/kinetics and antimicrobial susceptibility testing) of the activated sludge could provide experimental validation on the 16S rRNA gene-based findings. Overall, this work advanced the mechanistic understanding of how the antimicrobial TCS shapes the structure and diversity of activated sludge communities, which will be of great importance for managing and predicting microbial communities in engineered biological systems to provide better services to human society.

## Supplementary information

Figs. S1-3
