# Supplementary material for: Ecological processes underpinning microbial community structure during exposure to subinhibitory level of triclosan
Source: Sci Rep. 2019 Mar 14;9:4598. doi: 10.1038/s41598-019-40936-5 (PMC6418085; doi:10.1038/s41598-019-40936-5)
Supplement: Supplementary file 1 — Figs. S1-3 [file 41598_2019_40936_MOESM1_ESM.docx]

**SUPPLEMENTARY MATERIAL**

**Ecological processes underpinning microbial community structure during exposure to subinhibitory level of triclosan**

Seungdae Oh^1,^*, Donggeon Choi^1^, and Chang-Jun Cha^2^

^1^ Department of Civil Engineering, Kyung Hee University, Yongin-si, Gyeonggi-do, Republic of Korea

^2^ Department of Systems Biotechnology and Center for Antibiotic Resistome, Chung-Ang University, Anseong-si, Gyeonggi-do, Republic of Korea

* Corresponding author

Department of Civil Engineering

Kyung Hee University

1732 Deogyeong-daero, Giheung-gu, Yongin-si, Gyeonggi-do, Republic of Korea

Phone: +82 (031) 201-3664

Fax: +82 (031) 202-8854

E-mail: soh[@khu.ac.kr](mailto:kostas@ce.gatech.edu)


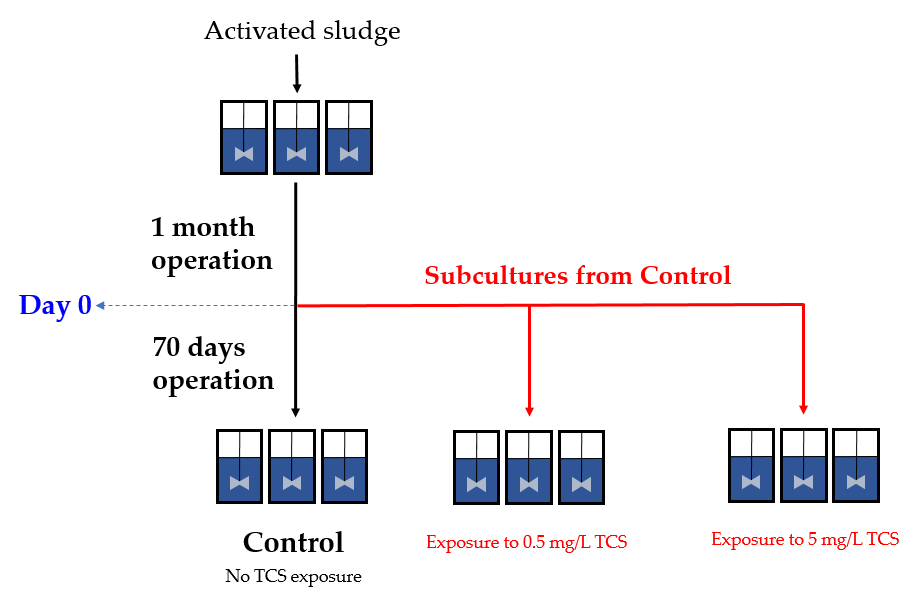


**Figure S1. Development of bioreactors.**


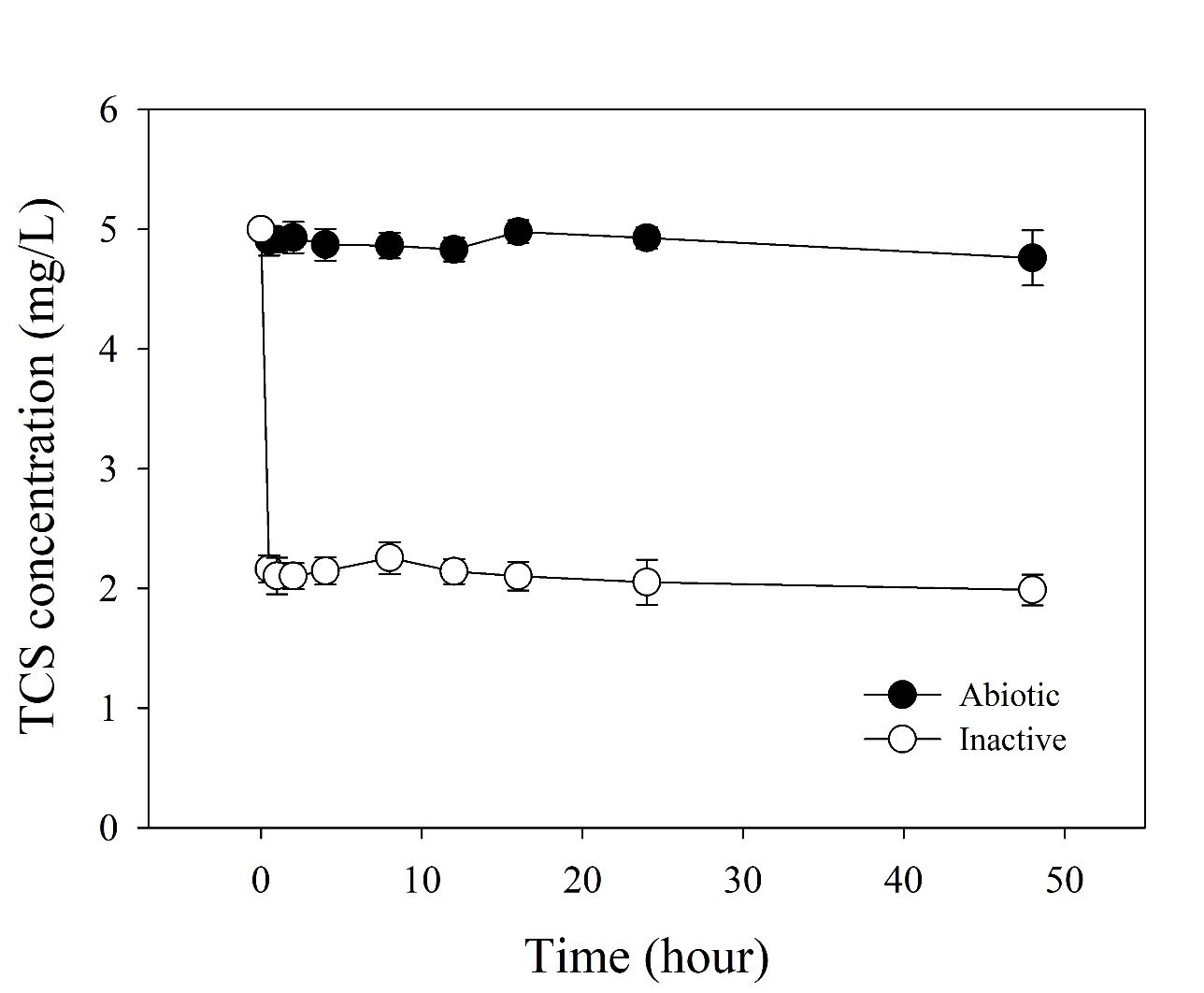


**Figure S2. TCS removal under abiotic and inactive conditions.** Closed circle represents abiotic condition without inoculum and open circle represents inactive inoculum by autoclaving.


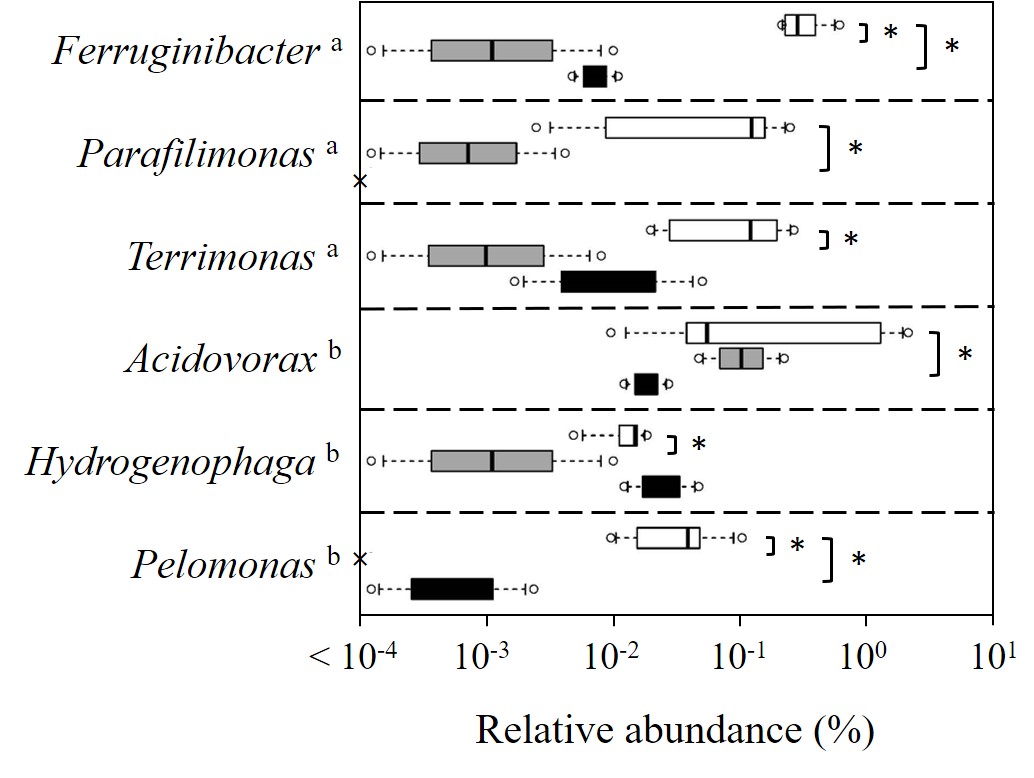


**Figure S3. Relative abundance of genera affiliated to *Chitinophagaceae* and *Comamonadaceae.*** The white, grey, and black colors denote the relative abundance of the control, TCS_3.5_, and TCS_56_ communities, respectively. The small circles represent outliers that are 1.5 × the interquartile range (IQR) lower than Q_1_ or 1.5 × IQR higher than Q_3_. The asterisk represents statistically-significant differential abundance (*P* < 0.05 by Mann-Whitney U test) between the TCS-exposed and control communities. The cross-marks represent those less than 10^-4^ in relative abundance (%). ^*^ Higher classification of a genus: ^a^ *Chitinophagaceae* and ^b^ *Comamonadaceae*.
